# 4-[4-(1*H*-Tetra­zol-5-yl)phen­oxy]benzaldehyde

**DOI:** 10.1107/S1600536811044254

**Published:** 2011-10-29

**Authors:** Jing Lu, Jiao Xu, Li-Wei Ni, Wei-Li Ma, Zhen-Ting Du

**Affiliations:** a College of Science, Northwest A&F University, Yangling Shaanxi 712100, People’s Republic of China

## Abstract

The asymmetric unit of the title compound, C_14_H_10_N_4_O_2_, contains two independent mol­ecules with similar structures. In one mol­ecule, the tetra­zole ring is oriented at dihedral angles of 17.71 (16) and 57.13 (17)°, respectively, to the central benzene ring and the terminal benzene ring; in the other mol­ecule, the corresponding dihedral angles are 16.46 (18) and 75.87 (18)°. Inter­molecular N—H⋯N hydrogen bonds and weak C—H⋯O and C—H⋯N hydrogen bonds occur in the crystal structure.

## Related literature

For the synthesis of 5-substituted 1*H*-tetra­zoles, see: Ostrovskii *et al.* (2008 ▶); Saikia & Phukan (2009 ▶); Nasrollahzadeh *et al.* (2009 ▶); Teimouri & Najafi Chermahini (2011 ▶). For related structures, see: Li *et al.* (2008 ▶); Hu *et al.* (2009 ▶); Xu *et al.* (2010 ▶); Deng *et al.* (2010 ▶).
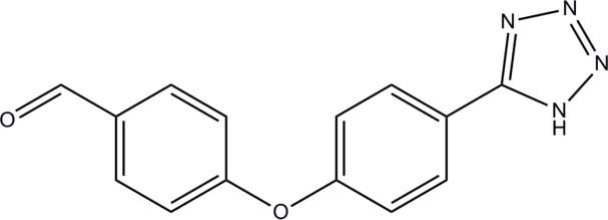


## Experimental

### 

#### Crystal data

C_14_H_10_N_4_O_2_
*M* *_r_* = 266.26
Triclinic, 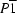

*a* = 9.854 (4) Å
*b* = 9.948 (4) Å
*c* = 14.139 (6) Å
α = 98.537 (4)°
β = 106.668 (4)°
γ = 99.737 (4)°
*V* = 1279.8 (9) Å^3^
*Z* = 4
Mo *K*α radiation
μ = 0.10 mm^−1^
*T* = 296 K
0.23 × 0.21 × 0.19 mm

#### Data collection

Bruker APEXII CCD diffractometer
9274 measured reflections
4683 independent reflections
2623 reflections with *I* > 2σ(*I*)
*R*_int_ = 0.035

#### Refinement

*R*[*F* ^2^ > 2σ(*F* ^2^)] = 0.056
*wR*(*F* ^2^) = 0.142
*S* = 1.01
4683 reflections
369 parameters
30 restraints
H atoms treated by a mixture of independent and constrained refinement
Δρ_max_ = 0.41 e Å^−3^
Δρ_min_ = −0.30 e Å^−3^

### 

Data collection: *APEX2* (Bruker, 2005 ▶); cell refinement: *SAINT* (Bruker, 2001 ▶); data reduction: *SAINT*; program(s) used to solve structure: *SHELXTL* (Sheldrick, 2008 ▶); program(s) used to refine structure: *SHELXTL*; molecular graphics: *SHELXTL*; software used to prepare material for publication: *SHELXTL*.

## Supplementary Material

Crystal structure: contains datablock(s) global, I, New_Global_Publ_Block. DOI: 10.1107/S1600536811044254/xu5357sup1.cif

Structure factors: contains datablock(s) I. DOI: 10.1107/S1600536811044254/xu5357Isup2.hkl

Supplementary material file. DOI: 10.1107/S1600536811044254/xu5357Isup3.cdx

Supplementary material file. DOI: 10.1107/S1600536811044254/xu5357Isup4.cml

Additional supplementary materials:  crystallographic information; 3D view; checkCIF report

## Figures and Tables

**Table 1:** Hydrogen-bond geometry (Å, °)

*D*—H⋯*A*	*D*—H	H⋯*A*	*D*⋯*A*	*D*—H⋯*A*
N1—H1*N*⋯N8^i^	0.91 (3)	1.94 (3)	2.849 (4)	174 (2)
N5—H2*N*⋯N4^ii^	0.90 (3)	2.03 (3)	2.924 (3)	176 (3)
C4—H4⋯O4	0.93	2.54	3.420 (4)	158
C17—H17⋯N3^ii^	0.93	2.58	3.365 (4)	143
C23—H23⋯O2^iii^	0.93	2.42	3.262 (6)	151

Symmetry codes: (i) 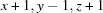; (ii) 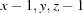; (iii) 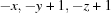.

**Fig. 1.**
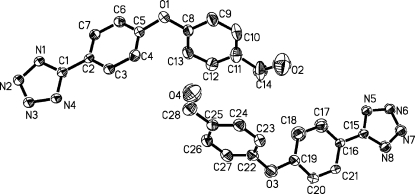
The molecular structure of the title compound with the atom numbering scheme. Displacement ellipsoids are drawn at the 30% probability level.

## supplementary crystallographic information

### Comment

Tetrazoles play a variety of roles in coordination chemistry, medicinal chemistry, materials chemistry etc. (Ostrovskii *et al.*, 2008). We intended to find a new way for preparation of tetrazoles using a new solid acid which is regarded as a green catalyst. In order to confirm the verity of the final product, the single crystal X-ray analysis was performed and the structure is reported here.

Compared with the other phenyltetrazole coplanar structures reported by Li *et al.* (2008) and Xu *et al.* (2010), there is torsion between tetrazole rings and neighboring phenyl rings, with a dihedral angle 17.7 (1)° and 16.4 (4)°, respectively.

### Experimental

A solution of 4-(4-formylphenoxy)benzonitrile (892 mg, 4 mmol) and sodium azide (780 mg, 12 mmol) in dry DMF (15 mL) at the presence of sulfuric acid on silica gel (5% load, 400 mg ) was heated to 80°C. When the reaction was completed, the solid acid was filtered, 2 mL water was added to the filtration, and then was extracted with ethyl acetate (20 mL × 3). The ethyl acetate layers were combined and washed by 20 mL water, and then 15 mL saturated sodium chloride and dried over anhydrous sodium sulfate. The solution was evaporated and the residue was separated on silica gel column chromatography with a gradient of petroleum ether and ethyl acetate as eluent to yield 570 mg the title compound. The compound was then dissolved in methanol, and colorless crystals were formed on slow evaporation at room temperature over one week.

### Refinement

The H1N and H2N atoms were located in a difference Fourier map and refined isotropically. Other H atoms were placed in calculated positions with C—H = 0.93 Å and refined using a riding model with *U*_iso_(H) = 1.2*U*_eq_(C).

### Crystal data

**Table:**

C_14_H_10_N_4_O_2_	*Z* = 4
*M**_r_* = 266.26	*F*(000) = 552
Triclinic, *P*1	*D*_x_ = 1.382 Mg m^−^^3^
Hall symbol: -P 1	Mo *K*α radiation, λ = 0.71073 Å
*a* = 9.854 (4) Å	Cell parameters from 1475 reflections
*b* = 9.948 (4) Å	θ = 2.2–21.0°
*c* = 14.139 (6) Å	µ = 0.10 mm^−^^1^
α = 98.537 (4)°	*T* = 296 K
β = 106.668 (4)°	Block, colourless
γ = 99.737 (4)°	0.23 × 0.21 × 0.19 mm
*V* = 1279.8 (9) Å^3^	

### Data collection

**Table:**

Bruker APEXII CCD diffractometer	2623 reflections with *I* > 2σ(*I*)
Radiation source: fine-focus sealed tube	*R*_int_ = 0.035
graphite	θ_max_ = 25.5°, θ_min_ = 2.2°
φ and ω scans	*h* = −11→11
9274 measured reflections	*k* = −10→12
4683 independent reflections	*l* = −17→17

### Refinement

**Table:**

Refinement on *F*^2^	Primary atom site location: structure-invariant direct methods
Least-squares matrix: full	Secondary atom site location: difference Fourier map
*R*[*F*^2^ > 2σ(*F*^2^)] = 0.056	Hydrogen site location: inferred from neighbouring sites
*wR*(*F*^2^) = 0.142	H atoms treated by a mixture of independent and constrained refinement
*S* = 1.01	*w* = 1/[σ^2^(*F*_o_^2^) + (0.0462*P*)^2^ + 0.4163*P*] where *P* = (*F*_o_^2^ + 2*F*_c_^2^)/3
4683 reflections	(Δ/σ)_max_ < 0.001
369 parameters	Δρ_max_ = 0.41 e Å^−^^3^
30 restraints	Δρ_min_ = −0.30 e Å^−^^3^

### Special details

**Table:**

Geometry. All esds (except the esd in the dihedral angle between two l.s. planes) are estimated using the full covariance matrix. The cell esds are taken into account individually in the estimation of esds in distances, angles and torsion angles; correlations between esds in cell parameters are only used when they are defined by crystal symmetry. An approximate (isotropic) treatment of cell esds is used for estimating esds involving l.s. planes.
Refinement. Refinement of F^2^ against ALL reflections. The weighted R-factor wR and goodness of fit S are based on F^2^, conventional R-factors R are based on F, with F set to zero for negative F^2^. The threshold expression of F^2^ > 2sigma(F^2^) is used only for calculating R-factors(gt) etc. and is not relevant to the choice of reflections for refinement. R-factors based on F^2^ are statistically about twice as large as those based on F, and R- factors based on ALL data will be even larger.

### Fractional atomic coordinates and isotropic or equivalent isotropic displacement parameters (Å^2^)

**Table:**

	*x*	*y*	*z*	*U*_iso_*/*U*_eq_	
C1	0.8863 (3)	0.1795 (3)	1.0915 (2)	0.0414 (7)	
C2	0.7457 (3)	0.1392 (3)	1.0126 (2)	0.0415 (7)	
C3	0.6554 (3)	0.2318 (3)	1.0004 (2)	0.0514 (8)	
H3	0.6862	0.3205	1.0416	0.062*	
C4	0.5208 (3)	0.1945 (3)	0.9283 (2)	0.0602 (8)	
H4	0.4598	0.2568	0.9209	0.072*	
C5	0.4773 (3)	0.0638 (3)	0.8672 (2)	0.0543 (8)	
C6	0.5645 (3)	−0.0298 (3)	0.8772 (2)	0.0597 (8)	
H6	0.5335	−0.1177	0.8349	0.072*	
C7	0.6986 (3)	0.0072 (3)	0.9504 (2)	0.0533 (8)	
H7	0.7580	−0.0564	0.9583	0.064*	
C8	0.3025 (3)	0.0912 (3)	0.7196 (2)	0.0558 (8)	
C9	0.1567 (3)	0.0772 (4)	0.6724 (3)	0.0728 (10)	
H9	0.0880	0.0209	0.6914	0.087*	
C10	0.1142 (4)	0.1482 (4)	0.5964 (3)	0.0820 (11)	
H10	0.0155	0.1401	0.5647	0.098*	
C11	0.2142 (4)	0.2309 (4)	0.5662 (3)	0.0718 (9)	
C12	0.3588 (4)	0.2389 (3)	0.6127 (2)	0.0668 (9)	
H12	0.4274	0.2921	0.5918	0.080*	
C13	0.4051 (3)	0.1701 (3)	0.6898 (2)	0.0629 (9)	
H13	0.5037	0.1769	0.7208	0.075*	
C14	0.1805 (5)	0.3200 (5)	0.4876 (3)	0.1064 (14)	
H14	0.2555	0.3725	0.4720	0.128*	
C15	−0.0013 (3)	0.7097 (3)	0.1508 (2)	0.0430 (7)	
C16	0.1047 (3)	0.7403 (3)	0.2513 (2)	0.0455 (7)	
C17	0.1222 (4)	0.6369 (3)	0.3065 (2)	0.0667 (9)	
H17	0.0665	0.5468	0.2782	0.080*	
C18	0.2201 (4)	0.6649 (3)	0.4020 (2)	0.0747 (11)	
H18	0.2318	0.5936	0.4373	0.090*	
C19	0.3005 (3)	0.7972 (3)	0.4453 (2)	0.0587 (8)	
C20	0.2850 (3)	0.9026 (3)	0.3926 (2)	0.0606 (9)	
H20	0.3397	0.9927	0.4219	0.073*	
C21	0.1884 (3)	0.8738 (3)	0.2964 (2)	0.0530 (8)	
H21	0.1788	0.9451	0.2609	0.064*	
C22	0.4087 (4)	0.7458 (3)	0.6065 (2)	0.0612 (9)	
C23	0.2899 (4)	0.7028 (3)	0.6363 (3)	0.0678 (9)	
H23	0.2027	0.7280	0.6082	0.081*	
C24	0.3016 (3)	0.6222 (3)	0.7079 (3)	0.0627 (9)	
H24	0.2217	0.5927	0.7282	0.075*	
C25	0.4307 (3)	0.5845 (3)	0.7501 (2)	0.0551 (8)	
C26	0.5488 (4)	0.6304 (3)	0.7202 (3)	0.0644 (9)	
H26	0.6365	0.6065	0.7489	0.077*	
C27	0.5387 (4)	0.7110 (3)	0.6487 (3)	0.0663 (9)	
H27	0.6189	0.7416	0.6290	0.080*	
C28	0.4455 (4)	0.4991 (3)	0.8268 (3)	0.0734 (10)	
H28	0.5360	0.4795	0.8538	0.088*	
N1	0.9674 (3)	0.0935 (3)	1.12958 (18)	0.0499 (6)	
N2	1.0895 (3)	0.1684 (2)	1.20221 (19)	0.0581 (7)	
N3	1.0818 (3)	0.2977 (2)	1.20764 (19)	0.0584 (7)	
N4	0.9565 (2)	0.3084 (2)	1.13952 (18)	0.0503 (6)	
N5	−0.0625 (3)	0.5829 (2)	0.09329 (18)	0.0466 (6)	
N6	−0.1563 (3)	0.5921 (2)	0.00568 (18)	0.0545 (6)	
N7	−0.1514 (3)	0.7233 (2)	0.00983 (18)	0.0565 (7)	
N8	−0.0556 (3)	0.7999 (2)	0.09919 (18)	0.0520 (6)	
O1	0.3385 (2)	0.0200 (2)	0.79639 (17)	0.0707 (7)	
O2	0.0601 (4)	0.3220 (4)	0.4469 (3)	0.1609 (14)	
O3	0.4057 (2)	0.8339 (2)	0.53920 (17)	0.0789 (7)	
O4	0.3497 (3)	0.4526 (3)	0.8575 (2)	0.0967 (9)	
H2N	−0.053 (3)	0.499 (3)	0.107 (2)	0.058 (9)*	
H1N	0.954 (3)	−0.001 (3)	1.117 (2)	0.081 (11)*	

### Atomic displacement parameters (Å^2^)

**Table:**

	*U*^11^	*U*^22^	*U*^33^	*U*^12^	*U*^13^	*U*^23^
C1	0.0474 (17)	0.0281 (14)	0.0483 (17)	0.0103 (13)	0.0123 (14)	0.0106 (13)
C2	0.0441 (16)	0.0303 (14)	0.0494 (17)	0.0101 (12)	0.0107 (14)	0.0121 (12)
C3	0.0529 (19)	0.0350 (15)	0.0599 (19)	0.0138 (14)	0.0082 (16)	0.0058 (14)
C4	0.0524 (19)	0.0473 (18)	0.076 (2)	0.0198 (16)	0.0077 (17)	0.0148 (17)
C5	0.0447 (18)	0.0490 (18)	0.0581 (19)	0.0046 (15)	0.0008 (15)	0.0153 (15)
C6	0.065 (2)	0.0373 (16)	0.061 (2)	0.0086 (15)	0.0021 (17)	0.0003 (14)
C7	0.0558 (19)	0.0388 (16)	0.0597 (19)	0.0161 (14)	0.0080 (16)	0.0082 (14)
C8	0.053 (2)	0.0499 (18)	0.0533 (19)	0.0130 (16)	0.0014 (16)	0.0058 (15)
C9	0.046 (2)	0.094 (3)	0.073 (2)	0.0132 (19)	0.0095 (18)	0.024 (2)
C10	0.052 (2)	0.119 (3)	0.070 (2)	0.031 (2)	0.004 (2)	0.022 (2)
C11	0.069 (2)	0.093 (3)	0.060 (2)	0.041 (2)	0.0175 (19)	0.0136 (18)
C12	0.071 (2)	0.069 (2)	0.061 (2)	0.0207 (19)	0.0215 (19)	0.0127 (18)
C13	0.0494 (19)	0.068 (2)	0.063 (2)	0.0149 (17)	0.0072 (17)	0.0101 (18)
C14	0.090 (3)	0.133 (3)	0.086 (3)	0.070 (2)	−0.007 (2)	0.011 (2)
C15	0.0487 (17)	0.0287 (14)	0.0513 (17)	0.0090 (13)	0.0157 (14)	0.0082 (13)
C16	0.0487 (17)	0.0318 (15)	0.0509 (17)	0.0096 (13)	0.0088 (14)	0.0062 (13)
C17	0.083 (2)	0.0308 (16)	0.062 (2)	−0.0002 (16)	−0.0051 (18)	0.0075 (15)
C18	0.096 (3)	0.0411 (18)	0.062 (2)	0.0041 (18)	−0.009 (2)	0.0154 (16)
C19	0.058 (2)	0.0500 (19)	0.0533 (19)	0.0019 (16)	0.0007 (16)	0.0103 (16)
C20	0.0534 (19)	0.0372 (17)	0.073 (2)	−0.0035 (14)	0.0022 (17)	0.0066 (16)
C21	0.0505 (18)	0.0336 (15)	0.067 (2)	0.0050 (14)	0.0064 (16)	0.0148 (14)
C22	0.059 (2)	0.0532 (19)	0.0505 (19)	−0.0055 (17)	−0.0011 (17)	0.0063 (16)
C23	0.053 (2)	0.065 (2)	0.071 (2)	0.0110 (17)	0.0019 (18)	0.0117 (19)
C24	0.053 (2)	0.061 (2)	0.071 (2)	0.0094 (17)	0.0178 (17)	0.0098 (18)
C25	0.058 (2)	0.0457 (18)	0.0545 (19)	0.0123 (16)	0.0111 (17)	0.0031 (15)
C26	0.053 (2)	0.062 (2)	0.070 (2)	0.0134 (17)	0.0078 (18)	0.0096 (18)
C27	0.051 (2)	0.072 (2)	0.068 (2)	0.0037 (17)	0.0157 (18)	0.0101 (19)
C28	0.085 (3)	0.060 (2)	0.080 (3)	0.026 (2)	0.030 (2)	0.011 (2)
N1	0.0518 (16)	0.0326 (14)	0.0574 (16)	0.0109 (12)	0.0044 (13)	0.0101 (12)
N2	0.0553 (16)	0.0395 (14)	0.0648 (17)	0.0116 (12)	−0.0032 (13)	0.0093 (12)
N3	0.0588 (17)	0.0371 (14)	0.0655 (17)	0.0109 (12)	−0.0001 (14)	0.0086 (12)
N4	0.0510 (15)	0.0313 (13)	0.0593 (15)	0.0086 (11)	0.0048 (13)	0.0077 (11)
N5	0.0557 (15)	0.0295 (13)	0.0505 (15)	0.0124 (12)	0.0078 (12)	0.0106 (11)
N6	0.0652 (17)	0.0411 (14)	0.0505 (16)	0.0131 (12)	0.0072 (13)	0.0103 (12)
N7	0.0693 (17)	0.0396 (14)	0.0541 (16)	0.0144 (13)	0.0083 (14)	0.0107 (12)
N8	0.0654 (16)	0.0334 (13)	0.0548 (15)	0.0156 (12)	0.0108 (13)	0.0132 (12)
O1	0.0513 (13)	0.0620 (14)	0.0781 (16)	−0.0014 (11)	−0.0063 (12)	0.0215 (12)
O2	0.142 (3)	0.222 (4)	0.138 (3)	0.066 (3)	0.038 (3)	0.081 (3)
O3	0.0761 (16)	0.0672 (15)	0.0615 (15)	−0.0176 (12)	−0.0099 (13)	0.0190 (12)
O4	0.129 (2)	0.0865 (19)	0.111 (2)	0.0483 (18)	0.071 (2)	0.0389 (17)

### Geometric parameters (Å, °)

**Table:**

C1—N4	1.325 (3)	C16—C21	1.388 (4)
C1—N1	1.335 (3)	C17—C18	1.373 (4)
C1—C2	1.455 (4)	C17—H17	0.9300
C2—C3	1.381 (3)	C18—C19	1.366 (4)
C2—C7	1.389 (4)	C18—H18	0.9300
C3—C4	1.372 (4)	C19—C20	1.378 (4)
C3—H3	0.9300	C19—O3	1.381 (3)
C4—C5	1.373 (4)	C20—C21	1.374 (4)
C4—H4	0.9300	C20—H20	0.9300
C5—C6	1.366 (4)	C21—H21	0.9300
C5—O1	1.396 (3)	C22—C27	1.375 (4)
C6—C7	1.374 (4)	C22—C23	1.378 (4)
C6—H6	0.9300	C22—O3	1.384 (4)
C7—H7	0.9300	C23—C24	1.374 (4)
C8—C13	1.375 (4)	C23—H23	0.9300
C8—C9	1.372 (4)	C24—C25	1.381 (4)
C8—O1	1.378 (3)	C24—H24	0.9300
C9—C10	1.377 (4)	C25—C26	1.378 (4)
C9—H9	0.9300	C25—C28	1.464 (4)
C10—C11	1.380 (5)	C26—C27	1.373 (4)
C10—H10	0.9300	C26—H26	0.9300
C11—C12	1.369 (4)	C27—H27	0.9300
C11—C14	1.520 (5)	C28—O4	1.200 (4)
C12—C13	1.381 (4)	C28—H28	0.9300
C12—H12	0.9300	N1—N2	1.349 (3)
C13—H13	0.9300	N1—H1N	0.91 (3)
C14—O2	1.166 (4)	N2—N3	1.294 (3)
C14—H14	0.9300	N3—N4	1.363 (3)
C15—N8	1.325 (3)	N5—N6	1.343 (3)
C15—N5	1.332 (3)	N5—H2N	0.90 (3)
C15—C16	1.455 (4)	N6—N7	1.291 (3)
C16—C17	1.385 (4)	N7—N8	1.362 (3)
			
N4—C1—N1	107.5 (2)	C18—C17—H17	119.4
N4—C1—C2	126.2 (2)	C16—C17—H17	119.4
N1—C1—C2	126.3 (2)	C19—C18—C17	120.1 (3)
C3—C2—C7	119.0 (3)	C19—C18—H18	119.9
C3—C2—C1	119.8 (2)	C17—C18—H18	119.9
C7—C2—C1	121.1 (2)	C18—C19—C20	120.1 (3)
C4—C3—C2	120.8 (3)	C18—C19—O3	124.1 (3)
C4—C3—H3	119.6	C20—C19—O3	115.7 (3)
C2—C3—H3	119.6	C21—C20—C19	119.7 (3)
C5—C4—C3	119.0 (3)	C21—C20—H20	120.1
C5—C4—H4	120.5	C19—C20—H20	120.1
C3—C4—H4	120.5	C20—C21—C16	121.2 (3)
C6—C5—C4	121.5 (3)	C20—C21—H21	119.4
C6—C5—O1	117.8 (3)	C16—C21—H21	119.4
C4—C5—O1	120.6 (3)	C27—C22—C23	120.9 (3)
C5—C6—C7	119.3 (3)	C27—C22—O3	117.3 (3)
C5—C6—H6	120.3	C23—C22—O3	121.6 (3)
C7—C6—H6	120.3	C24—C23—C22	119.3 (3)
C6—C7—C2	120.3 (3)	C24—C23—H23	120.4
C6—C7—H7	119.8	C22—C23—H23	120.4
C2—C7—H7	119.8	C23—C24—C25	120.7 (3)
C13—C8—C9	121.2 (3)	C23—C24—H24	119.6
C13—C8—O1	122.7 (3)	C25—C24—H24	119.6
C9—C8—O1	116.1 (3)	C24—C25—C26	119.1 (3)
C8—C9—C10	118.7 (3)	C24—C25—C28	121.5 (3)
C8—C9—H9	120.7	C26—C25—C28	119.4 (3)
C10—C9—H9	120.7	C27—C26—C25	120.9 (3)
C9—C10—C11	121.6 (3)	C27—C26—H26	119.5
C9—C10—H10	119.2	C25—C26—H26	119.5
C11—C10—H10	119.2	C26—C27—C22	119.2 (3)
C12—C11—C10	118.1 (3)	C26—C27—H27	120.4
C12—C11—C14	115.6 (4)	C22—C27—H27	120.4
C10—C11—C14	126.3 (4)	O4—C28—C25	124.9 (4)
C11—C12—C13	121.7 (3)	O4—C28—H28	117.5
C11—C12—H12	119.2	C25—C28—H28	117.5
C13—C12—H12	119.2	C1—N1—N2	109.5 (2)
C8—C13—C12	118.6 (3)	C1—N1—H1N	133 (2)
C8—C13—H13	120.7	N2—N1—H1N	117 (2)
C12—C13—H13	120.7	N3—N2—N1	106.1 (2)
O2—C14—C11	119.7 (5)	N2—N3—N4	110.5 (2)
O2—C14—H14	120.2	C1—N4—N3	106.4 (2)
C11—C14—H14	120.2	C15—N5—N6	109.9 (2)
N8—C15—N5	107.1 (2)	C15—N5—H2N	129.8 (18)
N8—C15—C16	127.5 (2)	N6—N5—H2N	120.1 (18)
N5—C15—C16	125.4 (2)	N7—N6—N5	106.0 (2)
C17—C16—C21	117.8 (3)	N6—N7—N8	110.5 (2)
C17—C16—C15	120.6 (2)	C15—N8—N7	106.5 (2)
C21—C16—C15	121.6 (3)	C8—O1—C5	118.2 (2)
C18—C17—C16	121.2 (3)	C19—O3—C22	119.9 (2)
			
N4—C1—C2—C3	−17.7 (4)	C19—C20—C21—C16	0.5 (5)
N1—C1—C2—C3	161.4 (3)	C17—C16—C21—C20	−0.1 (4)
N4—C1—C2—C7	163.8 (3)	C15—C16—C21—C20	178.0 (3)
N1—C1—C2—C7	−17.1 (4)	C27—C22—C23—C24	−1.0 (5)
C7—C2—C3—C4	0.3 (4)	O3—C22—C23—C24	−176.1 (3)
C1—C2—C3—C4	−178.3 (3)	C22—C23—C24—C25	0.1 (5)
C2—C3—C4—C5	−0.8 (5)	C23—C24—C25—C26	0.8 (5)
C3—C4—C5—C6	0.5 (5)	C23—C24—C25—C28	179.7 (3)
C3—C4—C5—O1	176.9 (3)	C24—C25—C26—C27	−0.8 (5)
C4—C5—C6—C7	0.3 (5)	C28—C25—C26—C27	−179.8 (3)
O1—C5—C6—C7	−176.1 (3)	C25—C26—C27—C22	−0.1 (5)
C5—C6—C7—C2	−0.9 (5)	C23—C22—C27—C26	1.0 (5)
C3—C2—C7—C6	0.6 (4)	O3—C22—C27—C26	176.3 (3)
C1—C2—C7—C6	179.1 (3)	C24—C25—C28—O4	1.9 (5)
C13—C8—C9—C10	−2.4 (5)	C26—C25—C28—O4	−179.2 (3)
O1—C8—C9—C10	178.8 (3)	N4—C1—N1—N2	0.0 (3)
C8—C9—C10—C11	0.8 (6)	C2—C1—N1—N2	−179.2 (3)
C9—C10—C11—C12	1.3 (5)	C1—N1—N2—N3	0.0 (3)
C9—C10—C11—C14	−176.4 (3)	N1—N2—N3—N4	−0.1 (3)
C10—C11—C12—C13	−1.8 (5)	N1—C1—N4—N3	−0.1 (3)
C14—C11—C12—C13	176.1 (3)	C2—C1—N4—N3	179.1 (3)
C9—C8—C13—C12	1.9 (5)	N2—N3—N4—C1	0.1 (3)
O1—C8—C13—C12	−179.4 (3)	N8—C15—N5—N6	−0.5 (3)
C11—C12—C13—C8	0.3 (5)	C16—C15—N5—N6	179.3 (3)
C12—C11—C14—O2	−177.7 (4)	C15—N5—N6—N7	0.3 (3)
C10—C11—C14—O2	0.0 (7)	N5—N6—N7—N8	−0.1 (3)
N8—C15—C16—C17	162.4 (3)	N5—C15—N8—N7	0.4 (3)
N5—C15—C16—C17	−17.4 (4)	C16—C15—N8—N7	−179.4 (3)
N8—C15—C16—C21	−15.7 (5)	N6—N7—N8—C15	−0.2 (3)
N5—C15—C16—C21	164.5 (3)	C13—C8—O1—C5	21.7 (4)
C21—C16—C17—C18	−0.8 (5)	C9—C8—O1—C5	−159.5 (3)
C15—C16—C17—C18	−178.9 (3)	C6—C5—O1—C8	−120.5 (3)
C16—C17—C18—C19	1.4 (6)	C4—C5—O1—C8	63.0 (4)
C17—C18—C19—C20	−1.0 (5)	C18—C19—O3—C22	−17.6 (5)
C17—C18—C19—O3	−177.2 (3)	C20—C19—O3—C22	166.1 (3)
C18—C19—C20—C21	0.1 (5)	C27—C22—O3—C19	128.5 (3)
O3—C19—C20—C21	176.6 (3)	C23—C22—O3—C19	−56.3 (4)

### Hydrogen-bond geometry (Å, °)

**Table:**

*D*—H···*A*	*D*—H	H···*A*	*D*···*A*	*D*—H···*A*
N1—H1N···N8^i^	0.91 (3)	1.94 (3)	2.849 (4)	174 (2)
N5—H2N···N4^ii^	0.90 (3)	2.03 (3)	2.924 (3)	176 (3)
C4—H4···O4	0.93	2.54	3.420 (4)	158
C17—H17···N3^ii^	0.93	2.58	3.365 (4)	143
C23—H23···O2^iii^	0.93	2.42	3.262 (6)	151

Symmetry codes: (i) *x*+1, *y*−1, *z*+1; (ii) *x*−1, *y*, *z*−1; (iii) −*x*, −*y*+1, −*z*+1.
